# Is universal antifungal prophylaxis mandatory in adults after lung transplantation? a metaanalysis of observational studies

**DOI:** 10.1186/2197-425X-3-S1-A1020

**Published:** 2015-10-01

**Authors:** K Pilarczyk, J Heckmann, H Carstens, C Draese, H Jakob, N Pizanis, M Kamler

**Affiliations:** West German Heart and Vascular Center Essen, University Hospital Essen, Department for Thoracic and Cardiovascular Surgery, Essen, Germany; University Hopsital Essen, Department for Anesthesiology and Intensive Care Medicine, Essen, Germany

## Introduction

Lung transplant recipients are at high risk of invasive fungal disease (IFD), particularly invasive aspergillosis and candidiasis. In contrast to liver transplant patients, no randomized controlled trials or international guidelines on antifungal prophylaxis in the lung transplant population exist.

## Objective

We performed a meta-analysis to determine whether antifungal prophylaxis reduces the rate of fungal infections after lung transplant.

## Methods

MEDLINE, EMBASE, CENTRAL/CCTR, Google Scholar, and reference lists of relevant articles were searched for full text articles in English from 1990-2014. Assessments for eligibility, relevance, study validity and data extraction were performed by two reviewers independently using prespecified criteria. The primary outcome was incidence of fungal infections (FI).

## Results

A total of six eligible observational studies with a total of 748 patients were identified (five comparing universal with no prophylaxis and one comparing targeted with no prophylaxis): 448 patients were treated with antifungal prophylaxis and 300 patients without prophylaxis served as controls. The pooled Odds ratio (OR) for fungal infection (62 FI in the intervention arm, 82 in the control group) was 0.234 [random effects model, 95% confidence interval (CI) 0.097-0.564, p = 0.001, z = -3.237]. Pooled studies were characterized by substantial heterogeneity (I^2^ 66.64%). As mortality and rate of complications of antifungal medication were only infrequently reported, a pooled analysis for these outcomes could not be performed.

## Conclusion

The present study suggests that universal antifungal prophylaxis reduces incidence of FI after lung transplant. However included studies are limited by small sample size, single center structure without randomization, mixed population (including heart transplant, single and double lung transplant) and heterogeneity due to variations in immunosuppression, type and duration of antifungal prophylaxis and incidence of fungal infection. Therefore, the optimal drug, dosage and duration of prophylaxis remain uncertain and there is a clear need for an adequately powered, prospective randomized controlled trial.

